# Bioinformatics analysis of genes related to ferroptosis in hepatic ischemia-reperfusion injury

**DOI:** 10.3389/fgene.2022.1072544

**Published:** 2022-12-02

**Authors:** Shuo Sun, Jianming Xue, Yunfei Guo, Jianling Li

**Affiliations:** The Affiliated Hospital of Chengde Medical University, Chengde, Hebei, China

**Keywords:** hepatic ischemia-reperfusion injury, ferroptosis, bioinformatics analysis, prediction of drug-gene interactions, MiRNA prediction

## Abstract

**Background:** Primary liver cancer is the sixth most commonly diagnosed cancer and the third leading cause of cancer death worldwide in 2020, and it ranks fifth in global incidence. Liver resection or liver transplantation are the two most prominent surgical procedures for treating primary liver cancer. Both inevitably result in HIRI, causing severe complications for patients and affecting their prognosis and quality of survival. Ferroptosis, a newly discovered mode of cell death, is closely related to HIRI. We used bioinformatics analysis to explore the relationship between the two further.

**Methods:** The GEO database dataset GSE112713 and the FerrDB database data were selected to use bioinformatic analysis methods (difference analysis, FRGs identification, GO analysis, KEGG analysis, PPI network construction and analysis, Hub gene screening with GO analysis and KEGG analysis, intergenic interaction prediction, drug-gene interaction prediction, miRNA prediction) for both for correlation analysis. The GEO database dataset GSE15480 was selected for preliminary validation of the screened Hub genes.

**Results:** We analysed the dataset GSE112713 for differential gene expression before and after hepatic ischemia-reperfusion and identified by FRGs, yielding 11 genes. These 11 genes were subjected to GO, and KEGG analyses, and PPI networks were constructed and analysed. We also screened these 11 genes again to obtain 5 Hub genes and performed GO analysis, KEGG analysis, intergenic interaction prediction, drug-gene interaction prediction, and miRNA prediction on these 5 Hub genes. Finally, we obtained preliminary validation of all these 5 Hub genes by dataset GSE15480.

**Conclusion:** There is a close relationship between HIRI and ferroptosis, and inhibition of ferroptosis can potentially be a new approach to mitigate HIRI treatment in the future.

## Introduction

**FIGURE 1:**
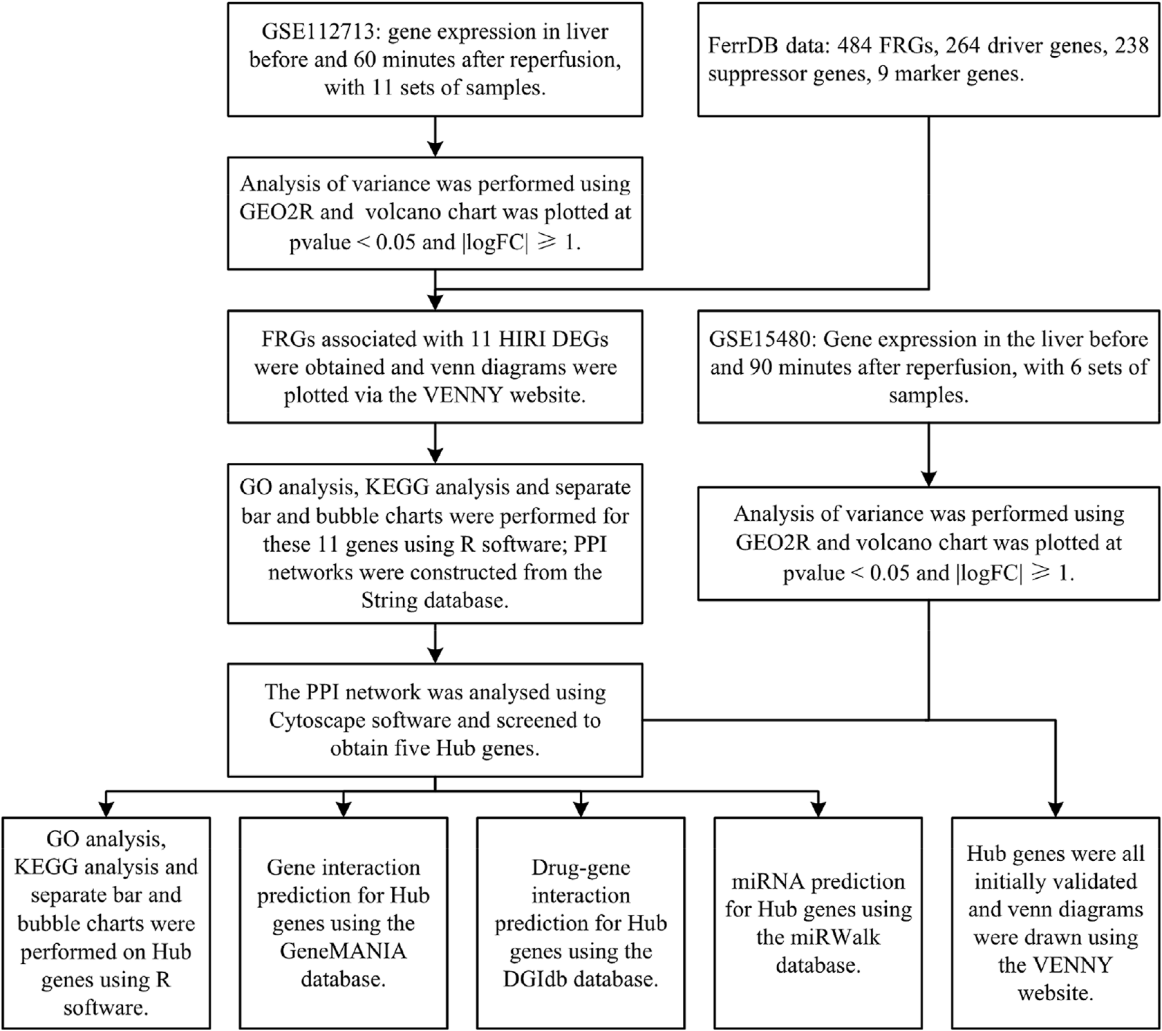
Technology roadmap: contains the overall design thinking for this study.

Cancer is the leading cause of human mortality and a significant barrier to increasing life expectancy in countries worldwide (Bray F et al., 2021). Primary liver cancer is the sixth most commonly diagnosed cancer and the third leading cause of cancer death worldwide in 2020, with approximately 906,000 new cases and 830,000 deaths (Sung H et al., 2021). In most areas, morbidity and mortality rates are two to three times higher for men than women (Sung H et al., 2021). Primary liver cancer ranks fifth in global incidence and second in male mortality (Sung H et al., 2021). Hepatectomy or liver transplantation are the two main surgical procedures used to treat primary liver cancer (Maki H et al., 2022), but hepatic ischemia-reperfusion injury (HIRI) is inevitable with hepatectomy or liver transplantation. HIRI may lead to liver dysfunction or even failure, causing severe inflammatory and stress reactions in patients. It can seriously affect various organs and systems, affecting their prognosis and quality of life (Nastos C et al., 2014). There are many studies on the pathophysiological mechanisms of HIRI, but they are still inconclusive and controversial. The concept of ferroptosis was first introduced by Dr Brent R. Stockwell in 2012 (Dixon S J et al., 2012). It is an iron-dependent mode of cell death caused by lipid peroxidation and massive accumulation of reactive oxygen radicals. It is a novel form of programmed cell death distinct from apoptosis, cell necrosis, and cell autophagy. Ferroptosis is associated with various biological contexts, from development to ageing, immunity and cancer^[6]^. Recent studies have shown that ferroptosis is closely related to the pathophysiology of many diseases, with ferroptosis being directly linked to ischemia-reperfusion injury (IRI) in many organs and, of course, HIRI being inextricably linked to ferroptosis (Li J. 2020; Stockwell B R. 2022). However, the relationship between HIRI and ferroptosis has been poorly studied, so we have used bioinformatics analysis to investigate the relationship between HIRI and ferroptosis (Figure 1) and provide a direction and foundation for future research.

## Materials and methods

### Data sources

The Gene Expression Omnibus (GEO) database is a public gene expression database created by the National Center for Biotechnology Information (NCBI) in the United States. It contains high-throughput gene expression data and microarray gene expression data (Edgar R. 2002). We selected the dataset GSE112713 for bioinformatic analysis of ferroptosis-related genes (FRGs). We selected one data set from GSE112713, a study on normothermic machine perfusion (NMP) inhibits proinflammatory responses in the liver and promotes regeneration: liver transplant patients, transplanted liver donors preserved by conventional refrigerated methods, and liver tissue extracted for microarray gene expression analysis before and 60 min after reperfusion at the end of preservation, for a total of 11 sets of samples (Jassem W et al., 2019).

The FerrDB database is the first in the world to integrate all data from the ferroptosis-related research literature in Pubmed (Zhou N et al., 2020), through which we downloaded FRGs and obtained a total of 484 FRGs, including driver genes 264, suppressor genes 238 and marker genes 9.

### Differential analysis of HIRI-related genes and mapping of volcano chart

We performed the differential analysis of gene expression data before and after liver reperfusion for 11 selected samples in the GSE112713 dataset by the GEO2R (Barrett T et al., 2012) online tool in the GEO database. We used the R software (version: 4.2.1) (R Core Team. 2022) ggplot2 package (version: 3.3.6) (H. Wickham. 2016) with *p*-value<0.05 and |logFC|≥1 for volcano chart plotting.

### FRGs identification and mapping of venn diagrams

We took the differentially expressed genes (DEGs) obtained for HIRI and intersected them with FRGs, and plotted them online *via* the VENNY (version: 2.1) website (Oliveros, 2007) to plot the venn diagram online.

### Gene ontology analysis and kyoto encyclopedia of genes and genomes analysis

We took the intersecting genes obtained from the DEGs of HIRI with FRGs obtained using the R software (version: 4.2.1) (R Core Team. 2022) clusterProfiler package (version: 4.4.4) (Wu T et al., 2021), the org.Hs.eg.db package (version: 3.15.0) (Marc C. 2022), the enrichplot package (version: 1.16.2) (Yu G. 2022), and ggplot2 package (version: 3.3.6) (H. Wickham. 2016) for GO analysis and KEGG analysis, and ranked the results of GO analysis and KEGG analysis from smallest to largest with *p*-value<0.05. The top ten results in GO analysis and the top thirty results in KEGG were selected and plotted as bar graphs and bubble plots respectively.

### Building protein-protein interaction networks

The String database (version: 11.5) is one of the most data-rich and widely used databases for studying protein interactions (Szklarczyk D et al., 2019). We put the obtained DEGs of HIRI with the intersecting genes obtained from FRGs through this database for PPI network construction.

### Cytoscape software analyses PPI networks and screens for hub genes

Cytoscape software (version: 3.9.1) is software that graphically displays networks and performs analysis and editing (Shannon P et al., 2003). CytoHubba is an APP within this software that screens Hub genes in PPI networks, and we used one of the most widely used MCC algorithms to process PPI networks and screen Hub genes (Chin C-H et al., 2014).

### Hub gene GO analysis and KEGG analysis

We performed GO and KEGG analyses on the screened Hub genes using the same methods described above.

### Predicting interactions between genes

The GeneMANIA database can help predict interactions between genes (Mostafavi S et al., 2008), and we used this database to predict interactions between genes associated with Hub genes.

### Prediction of pharmacogenetic interactions

The DGIdb database (version: 4.2.0) is a drug-gene interaction database that provides information on the association of genes with their known or potential drugs (Freshour S L et al., 2021), which we used to predict the drugs associated with them for Hub genes and visualised them using Cytoscape software (version: 3.9.1) for visualisation.

### miRNA prediction

The miRWalk database (version: 2.0) enables the prediction of gene-miRNA interactions (Sticht C et al., 2018). We used this database to make relevant predictions of miRNA expression for Hub genes and used it validated as a screening condition. The results were further screened and finally visualised using Cytoscape software (version: 3.9.1).

### Initial validation of the hub gene

We selected dataset GSE15480 from the GEO database to validate Hub genes. Dataset GSE15480 studies the global gene expression in deceased donor liver transplantation with ischemic preconditioning (Raza A et al., 2010). We selected six standards (no IPC) at preimplantation *versus* six standards (no IPC) at 90 min post-reperfusion for differential analysis by the GEO2R (Barrett T et al., 2012) online tool in the GEO database. We used the R software (version: 4.2.1) (R Core Team. 2022) ggplot2 package (version: 3.3.6) (H. Wickham. 2016) to *p*-value<0.05 and |logFC|≥1 for volcano chart plotting. Finally, the venn diagram was plotted online with FRGs, genes in Table 1, and Hub genes through the VENNY (version: 2.1) website (Oliveros, 2007) for initial validation of Hub genes.

**TABLE 1:** Symbols of DEGs for 11 HIRI-related FRGs, including the corresponding *p*-value, logFC, and FRGs.

Symbol	P-value	logFC	FRGs
PTGS2	0.000265	1.712139	Marker
TNFAIP3	0.00000453	1.300149	Driver
ATF3	0.000836635	1.430233	Driver
IL1B	0.00165	1.318098	Driver
IL6	0.0000000948	2.921581	Driver
MYCN	0.015	1.181727	Driver
MAP3K14	0.00121	1.120579	Driver
CDKN1A	0.000312	1.384527	Suppress
NR4A1	0.000369838	1.879928	Suppress
GDF15	0.00264	1.727425	Suppress
CREB5	0.000000594	1.51525	Suppress

## Results

### Differential analysis of HIRI-related genes and mapping of volcano chart

**FIGURE 2:**
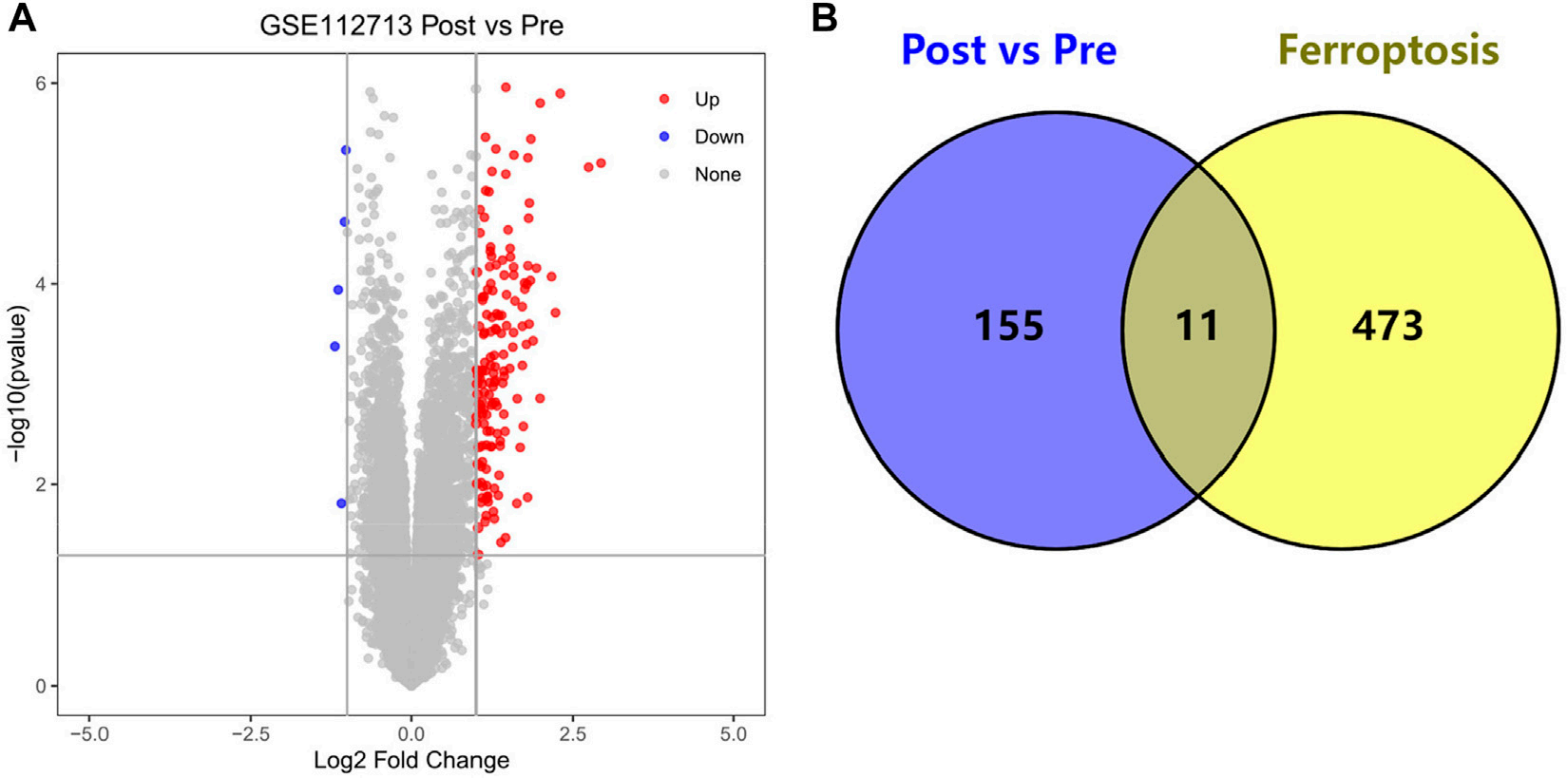
**(A)** Volcano chart mapped by 166 HIRI-related DEGs, **(B)** Venn chart of 166 “Post vs. Pre” DEGs with 484 “Ferroptosis” genes.

We obtained 166 HIRI-associated DEGs, of which 161 were up-regulated genes and 5 were down-regulated genes and mapped the volcano chart (Figure 2A).

### FRGs identification and mapping of venn diagrams

We obtained DEGs for 11 HIRI-associated FRGs (Table 1) and plotted venn chart (Figure 2B).

### GO analysis of HIRI-related FRGs

**FIGURE 3:**
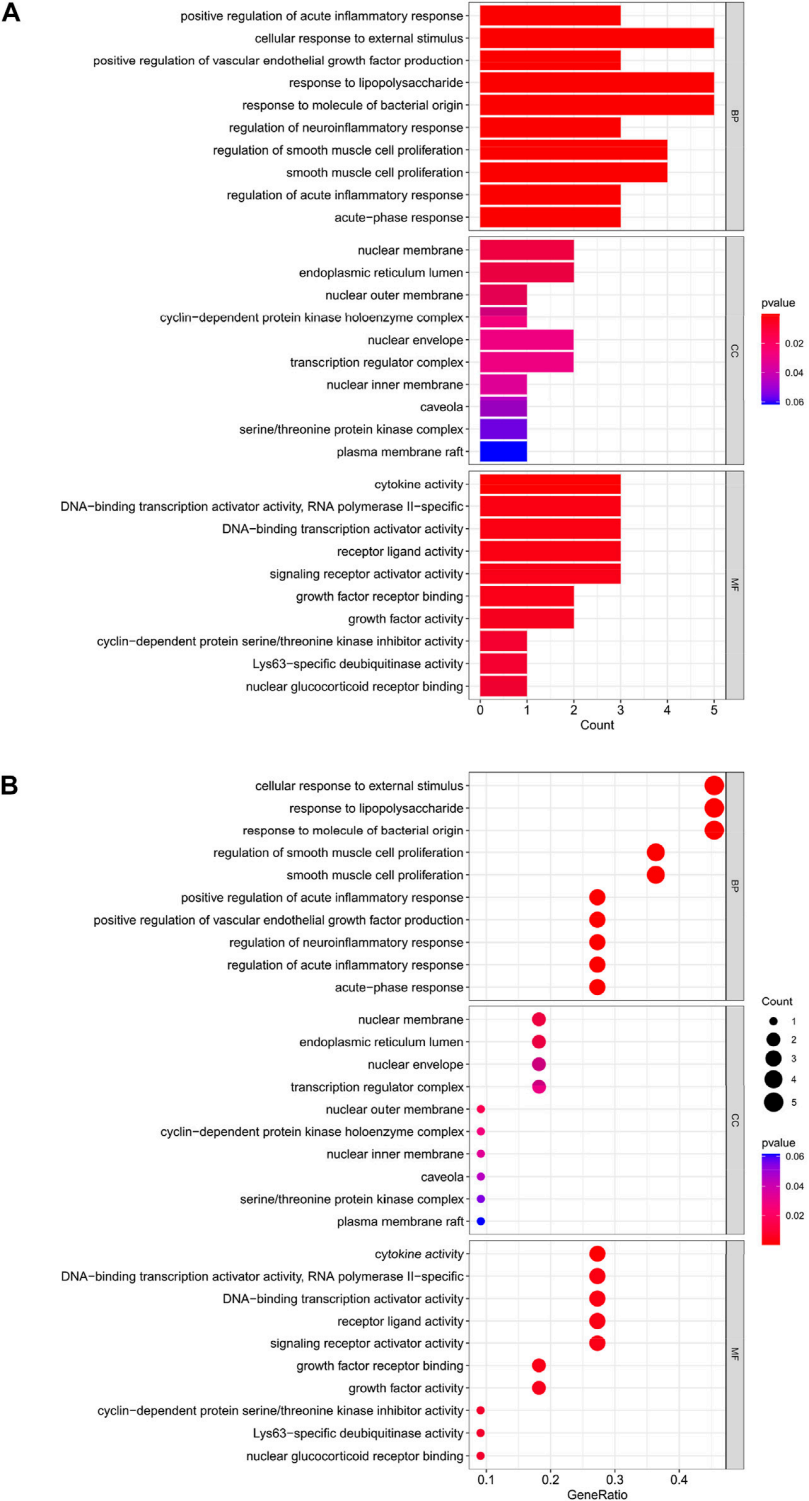
**(A)** Bar chart plotted against the results of GO analysis for the 11 genes in Table 1, **(B)** Bubble chart plotted against the results of GO analysis for the 11 genes in Table 1.

The top 10 results of the GO analysis with the smallest *p*-value for biological process (BP), cellular component (CC) and molecular function (MF) were selected and plotted in order of bar chart (Figure 3A) and bubble chart (Figure 3B). We can see from the figure that in BP, the central enrichment is in “cellular response to external stimulus”, “response to lipopolysaccharide” and “response to molecule of bacterial origin”; in CC, the central enrichment is in “nuclear membrane” and “endoplasmic reticulum lumen”; in MF, the central enrichment is in “cytokine activity”, “DNA-binding transcription activator activity, RNA polymerase II-specific”, “DNA-binding transcription activator activity”, “receptor ligand activity” and “signaling receptor activator activity".

### KEGG analysis of HIRI-associated FRGs

**FIGURE 4:**
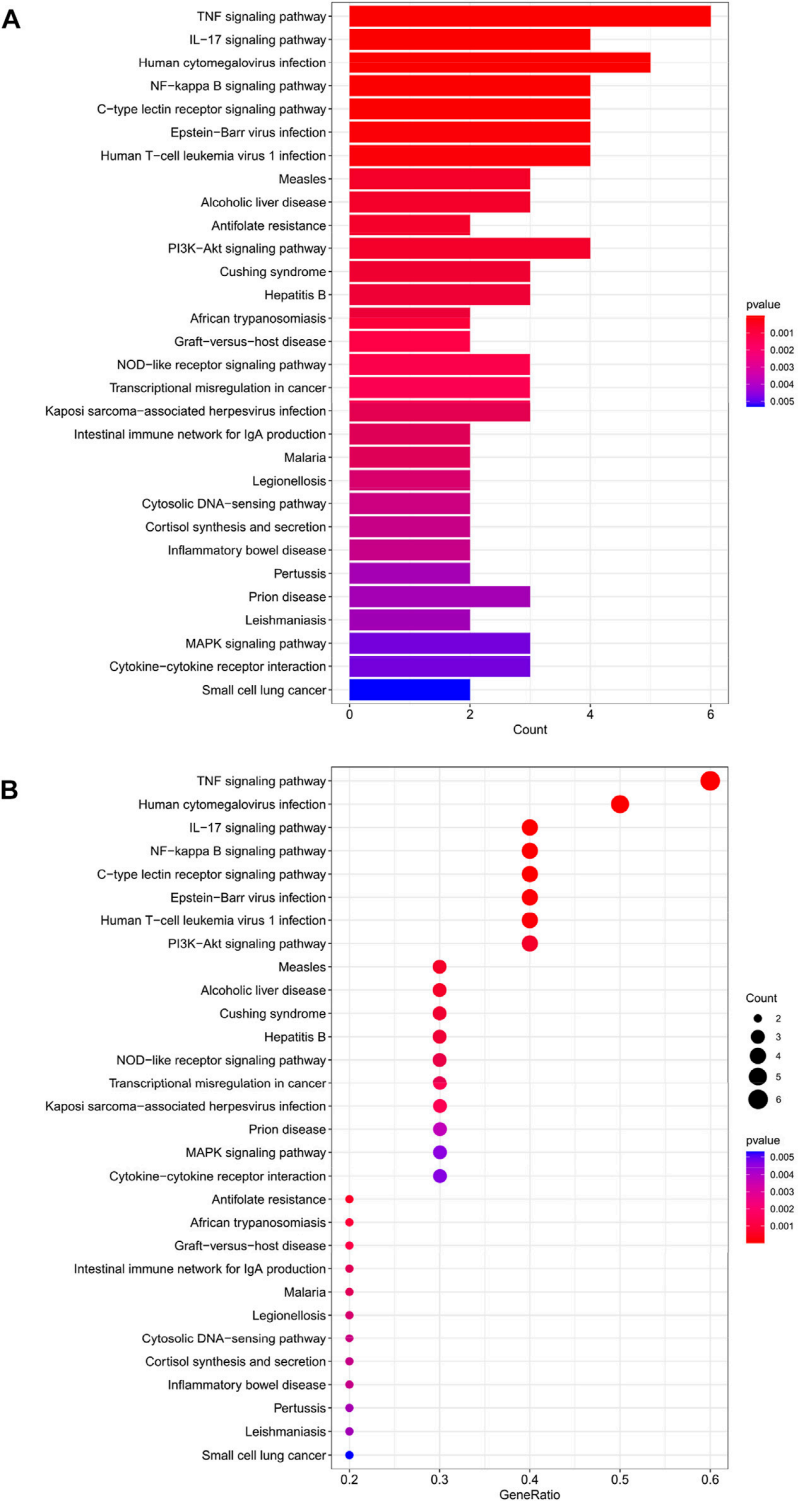
**(A)** Bar chart plotted against the results of KEGG analysis for the 11 genes in Table 1, **(B)** Bubble chart plotted against the results of KEGG analysis for the 11 genes in Table 1.

The top 30 analyses with the smallest *p*-value in the KEGG analysis were selected to plot a bar chart (Figure 4A) with a bubble chart (Figure 4B). The figure shows that there are eight main areas of enrichment, including “TNF signaling pathway” and “Human cytomegalovirus infection".

### HIRI-related FRGs build PPI networks

**FIGURE 5:**
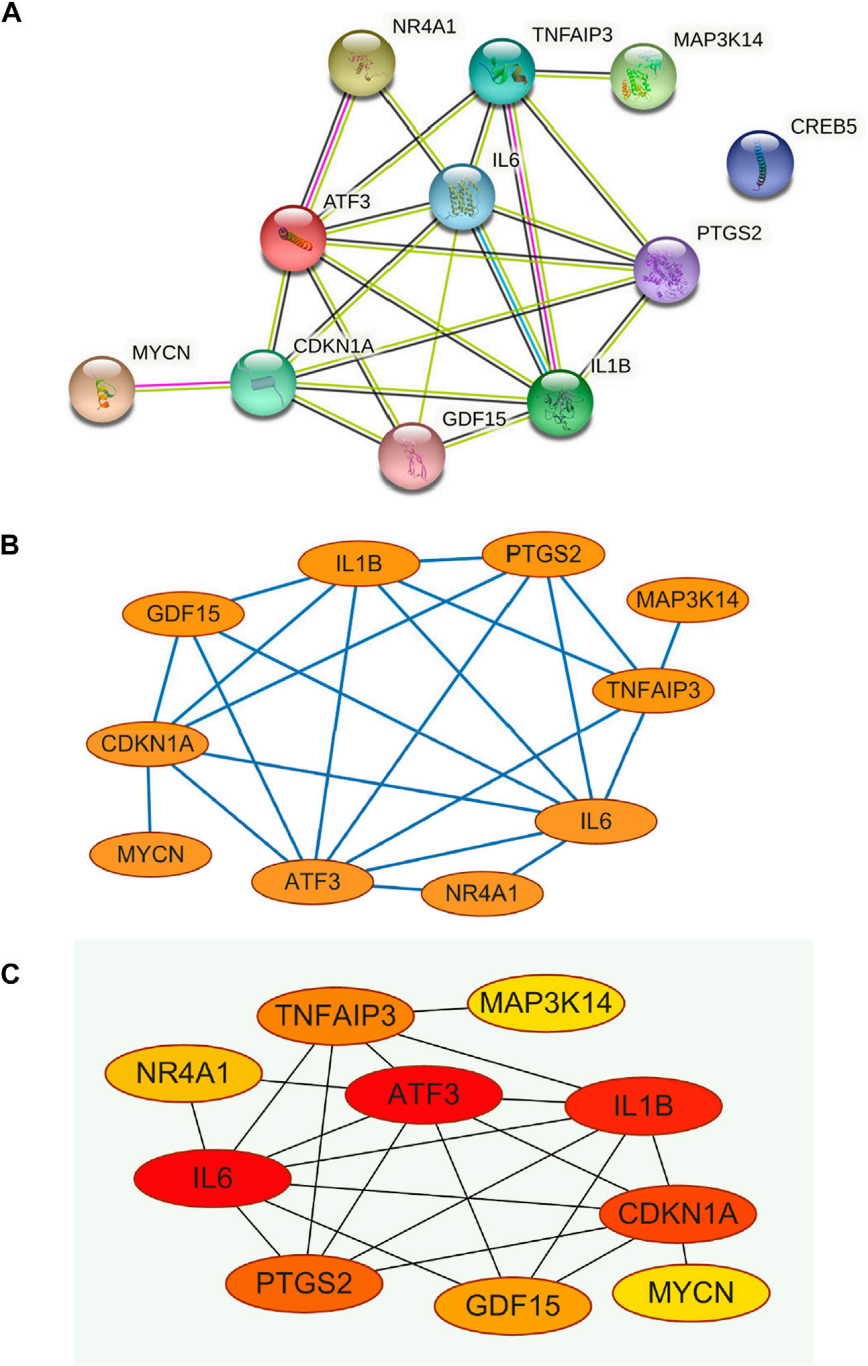
**(A)** Construction of PPI networks for the 11 genes in Table 1, **(B)** The 11 genes in Table 1 were analysed using Cytoscape software, **(C)** Analysis of the 11 genes in Table 1 using the MCC algorithm for the CytoHubba function in Cytoscape software yielded Hub Genes.

The PPI network was constructed for the 11 genes in Table 1 (Figure 5A), which has 11 nodes and 22 edges, with the “CREB5” gene not associated with any other gene.

### Cytoscape software analyses PPI networks to screen for hub genes

The constructed PPI network was analysed by the Cytoscape software CytoHubba function (Figure 5B and Figure 5C), and 11 genes were scored using the MCC algorithm (Table 2). The top 5 genes with the highest scores were selected as Hub genes, namely “ATF3”, “IL6”, “IL1B”, “CDKN1A”, and “PTGS2”.

**TABLE 2:** The 11 genes in Table 1 were scored by applying the MCC algorithm for the CytoHubba function in Cytoscape software.

Rank	Name	Score
1	ATF3	74
1	IL6	74
3	IL1B	72
4	CDKN1A	49
5	PTGS2	48
6	TNFAIP3	25
7	GDF15	24
8	NR4A1	2
9	MYCN	1
9	MAP3K14	1

### Hub gene GO analysis

**FIGURE 6:**
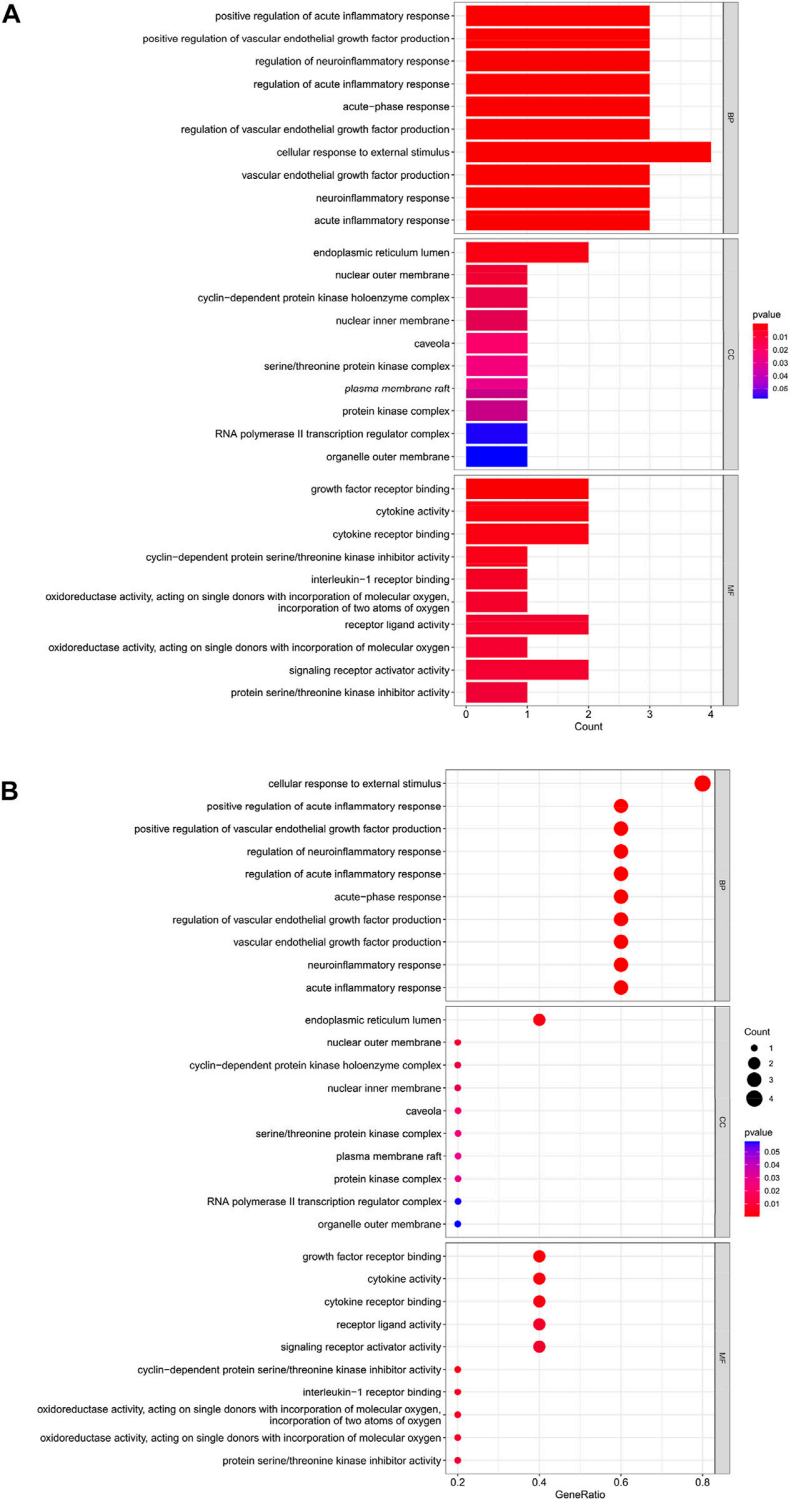
**(A)** Bar chart plotted against the results of GO analysis for the Hub gene, **(B)** Bubble chart plotted against the results of GO analysis for the Hub gene.

The top 10 results of the GO analysis with the smallest *p*-value for BP, CC and MF were selected and plotted in turn as a bar chart (Figure 6A) and bubble chart (Figure 6B). In the GO analysis of these 5 Hub genes, ten aspects were enriched in BP, including “cellular response to external stimulus”; in CC, the central enrichment was in 4 areas such as “endoplasmic reticulum lumen”; in MF, it is mainly enriched in “growth factor receptor binding”, “cytokine activity”, “cytokine receptor binding”, “receptor ligand activity”, “signaling receptor activator activity” these five aspects.

### Hub gene KEGG analysis

**FIGURE 7:**
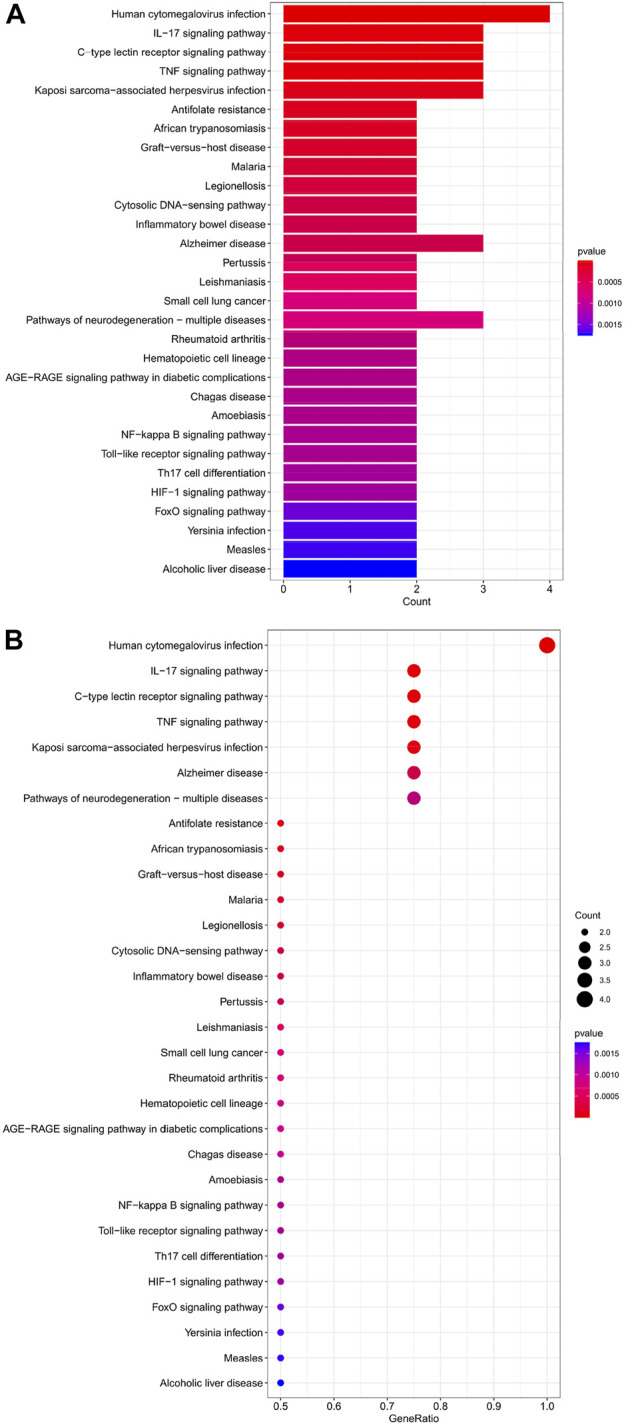
**(A)** Bar chart plotted against the results of KEGG analysis for the Hub gene, **(B)** Bubble chart plotted against the results of KEGG analysis for the Hub gene.

The top 30 analyses with the smallest *p*-value in the KEGG analysis were selected to plot a bar chart (Figure 7A) with a bubble chart (Figure 7B). As we can see from the figure, the central enrichment is in six areas, including “Human cytomegalovirus infection".

### Predicting interactions between genes

**FIGURE 8:**
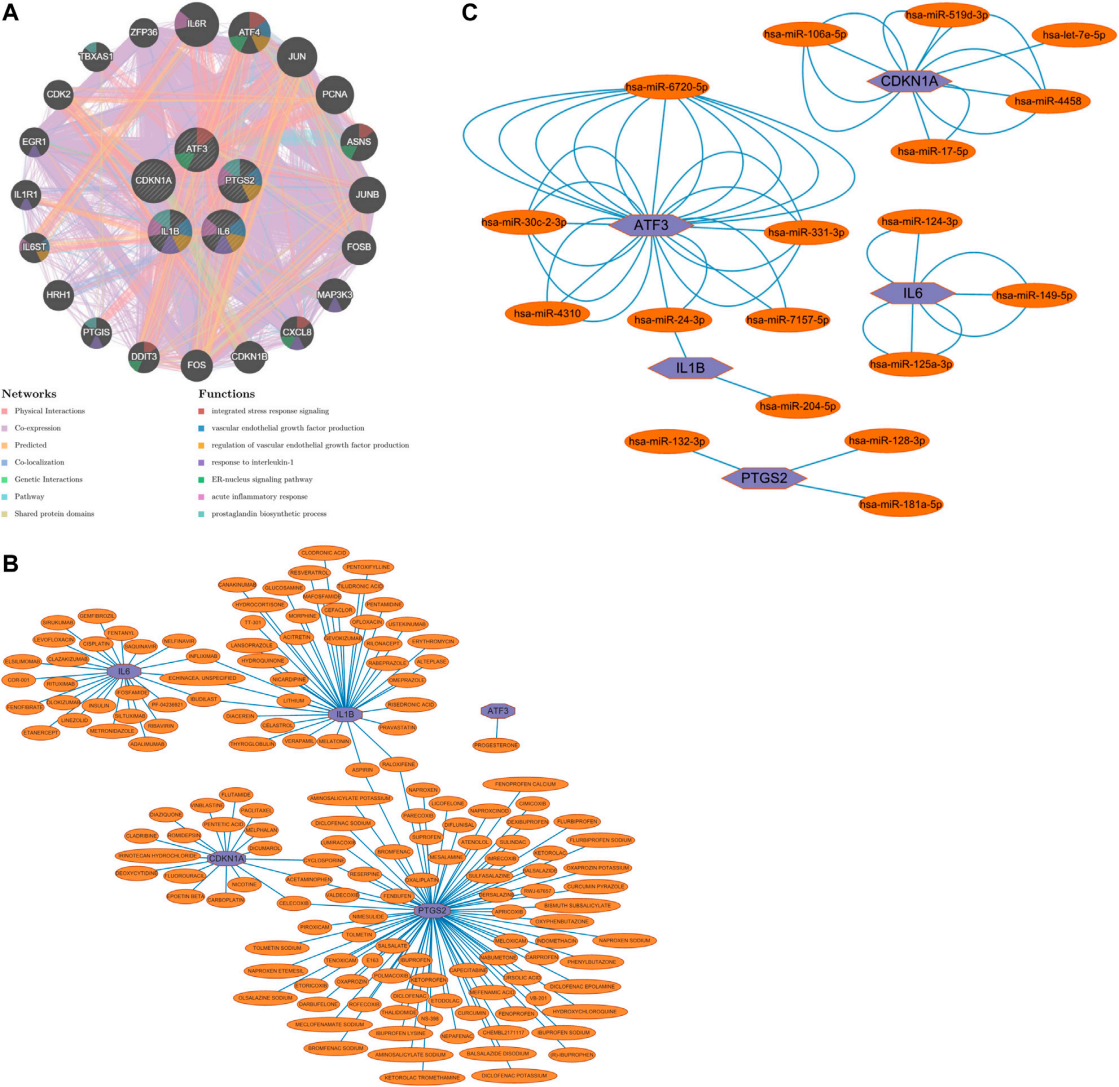
**(A)** Prediction of gene interactions on Hub Gene, **(B)** Prediction of drug-gene interactions on Hub Gene, **(C)** The miRNA expression prediction on Hub Gene.

The five Hub genes screened were predicted to interact with each other, and 20 genes were predicted to potentially interact with these five Hub genes (Figure 8A).

### Prediction of drug-gene interactions

Drug-gene interaction predictions for the five Hub genes screened predicted that one could act on ATF3, 25 on IL6, 37 on IL1B, 18 on CDKN1A and 83 on PTGS2; three on both IL6 and IL1B; two on both IL1B and PTGS2; and three on both CDKN1A and PTGS2 (Figure 8B).

### miRNA prediction

The five Hub genes screened were subjected to miRNA prediction. After screening the predicted miRNAs, there were six miRNAs potentially expressed by ATF3, two miRNAs potentially expressed by IL1B, three miRNAs potentially expressed by PTGS2, three miRNAs potentially expressed by IL6, and five miRNAs potentially expressed by CDKN1A, with one miRNA potentially expressed in common by ATF3 and IL1B (Figure 8C).

### Initial validation of the hub gene

**FIGURE 9:**
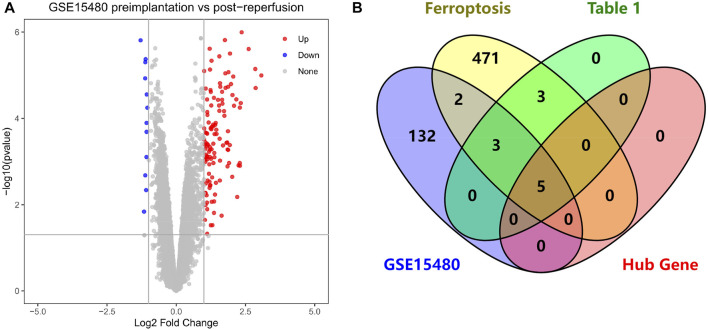
**(A)** Volcano chart mapped by 142 HIRI-related DEGs, **(B)** Venn chart of “Ferroptosis”, “Table 1”, “GSE15480”, “Hub Gene”.

We performed differential analysis on the dataset GSE15480 and obtained a total of 142 DEGs, including 129 up-regulated genes and 13 down-regulated genes, and mapped the volcano chart (Figure 9A). Compared with 484 genes in FRGs, 10 of them were initially validated, namely ATF3, TNFAIP3, IL1B, IL6, PTGS2, JUN, CDKN1A, GJA1, MAP3K14, NR4A1. Compared with 11 genes in Table 1, 8 were initially validated, namely ATF3, TNFAIP3, IL1B, IL6, PTGS2, CDKN1A, MAP3K14, and NR4A1. These were initially validated compared to the five Hub genes screened in Table 2 (Figure 9B).

## Discussion

HIRI is a complex pathophysiological process involving multiple factors acting together to exacerbate liver damage, dysfunction and increased structural damage when blood flow is restored after a period of insufficient or interrupted blood flow to the liver (Jaeschke H. 2003; Klune J R et al., 2010). Not only does it affect the physiological functions of the liver, leading to liver dysfunction or even failure in patients, but the severe stress and inflammatory response brought about by HIRI can cause acute dysfunction of other organs such as the heart, kidneys and lungs, and even acute multi-organ failure. HIRI is, therefore, not just a little complication but a severe complication with devastating systemic effects that severely affect patients’ prognosis and quality of survival (Nastos C et al., 2014). Whereas hepatectomy or liver transplantation is currently the predominant surgical procedure for treating primary liver cancer (Maki H et al., 2022), both inevitably result in HIRI. Therefore, the question of how to reduce or even avoid the harmful effects of HIRI has been a hot topic in clinical work and has not been effectively addressed.

The pathophysiological mechanisms of HIRI are currently known in terms of anaerobic metabolism, oxygen-free radical production, Ca^2+^ overload, Kupffer cell production, activation of the complement system, apoptosis and necrosis (Jaeschke H. 2003; Montalvo-Jave E E et al., 2008; Klune J R et al., 2010). However, many unknown pathophysiological mechanisms exist and have not been confirmed by research. The pathophysiological mechanisms of HIRI have therefore been controversial in clinical practice. Ferroptosis is a newly discovered mode of cell death and has been shown to play an essential role in HIRI (Li J. 2020; Stockwell B R. 2022). As a complement to the pathophysiological mechanisms of HIRI, there are fewer studies in this area, so we explored the relationship between the two in theory employing bioinformatics analysis.

Through our studies, we have found that differences in expression at the gene level occur in the liver before and after reperfusion after a period of ischaemia. We define *p*-value<0.05 and |logFC|≥1 as DEGs. These DEGs determine the differential expression of proteins. As seen from our plot of the volcano (Figure 2A), the vast majority of these DEGs are up-regulated genes. We, therefore, suggest that the differential proteins expressed by DEGs may be a cause of HIRI. Of these DEGs, 11 of these genes are FRGs (Figure 2B) (Table 1). Most of these FRGs are Driver genes (Zhou N et al., 2020), genes that drive ferroptosis, have a positive regulation of ferroptosis, and have a Mark gene (Zhou N et al., 2020) that does not regulate ferroptosis but which marks the occurrence of ferroptosis, reinforcing the existence of a strong link between ferroptosis and HIRI. In GO analysis of these 11 genes (Figure 3B) with KEGG analysis (Figure 4B), the necessary conditions were provided for us to validate ferroptosis with HIRI further. In the PPI network constructed for these 11 genes (Figure 5A), expression was strongly linked between genes, but the CREB5 gene was not linked to any other gene and could therefore be targeted for elimination in subsequent studies. We analysed the PPI network again using statistical methods (Figure 5B), narrowed it down and screened it again (Figure 5C), and screened for five Hub genes (Table 2). For the five Hub genes GO analysis (Figure 6B) with KEGG analysis in (Figure 7B), which is an essential basis for our following study. In a protein interaction prediction of Hub genes, there were 20 genes with which they interacted that were co-expressed in some way (Figure 8A), which provided a direction for our following study. We applied the study data from the dataset GSE15480 to provide a preliminary validation of our study. As shown in Figure 9B, the FRGs in this dataset overlap with the 11 genes screened in Table 1 and contained all of the Hub genes we screened. This further suggests that ferroptosis plays an essential role in HIRI with these five genes.

Activation transcription factor 3 (ATF3) is a basic leucine zipper (bZIP) DNA binding protein, a member of the ATF/CREB family of transcription factors, and the stress response induces widespread expression of ATF3 (Hai T et al., 1999). Furthermore, HIRI can lead to severe stress reactions in the organism. It has been shown that ATF3 promotes erastin-induced ferroptosis by suppressing system Xc^–^ (Wang L et al., 2020). ATF3 plays an essential role in ferroptosis (Dai C et al., 2020). In HIRI, ATF3 expression is significantly increased, significantly up-regulated and is a very important DEG (Zabala V et al., 2019; Zhong Y et al., 2019). In a comprehensive study of HIRI factor binding proteomics and transcriptomics (Huang S et al., 2019): it was shown that ATF3 is up-regulated by DEGs. This study reveals that ATF3 may be a core factor in HIRI and has potential clinical implications. In our study, ATF3 was the Hub gene, so we speculate that ferroptosis plays an essential role in HIRI in which ATF3 plays an important role.

Interleukin-6 (IL6) is a cytokine with pleiotropic activity that plays an essential role in the acute immune response of the body (Akira S et al., 1993). IL6 has been shown to be required for the induction of ATF3 (Hai T et al., 1999). In contrast, in bioinformatic analysis of ferroptosis in myocardial IRI (Wang Z et al., 2022), IL6 played an essential role in both. In our study, ATF3 and IL6 were screened as Hub genes in the ferroptosis bioinformatic analysis of HIRI; they were intricately linked in the PPI network and intergenic interaction prediction, and ATF3 and IL6 had the same maximum score when scored by the Cytoscape software CytoHubba functional MCC algorithm. We, therefore, hypothesise that ATF3 and IL6 have a central role in the induction of ferroptosis in HIRI. Also, the Hub genes screened in a raw letter analysis of IRI mechanisms in patients undergoing coronary artery bypass grafting (Shen Z et al., 2019) were identical to the Hub genes IL6, IL1B and PTGS2 predicted by our study, and PTGS2 is a Marker gene, and IL6 and IL1B are both Driver genes, thus reinforcing that ferroptosis has an essential role in HIRI.

How to mitigate or avoid the harm caused by HIRI to patients has always been a significant concern in clinical work and is currently a hot topic of research in clinical work. Ferroptosis, one of the pathophysiological mechanisms of HIRI, is beneficial in reducing HIRI by inhibiting the occurrence of ferroptosis. Numerous studies have shown (Kim K M et al., 2020; Yamada N et al., 2020; Liu H et al., 2021; Luo L et al., 2021) that some drugs, such as ferrostatin-1, alpha-tocopherol, deferoxamine, etc. or a low iron diet can reduce HIRI. Therefore, we have also done research in this area and predicted drug-gene interactions for the five Hub genes that were screened (Figure 8B). These predicted drugs could be a reference for future clinical work in targeting HIRI.

MicroRNA (miRNA) is a class of small, endogenous, non-coding RNA molecules of approximately 20–40 nucleotides in length (Saliminejad K et al., 2019). It has a compelling physiological role in regulating gene expression, controlling early development, cell proliferation, apoptosis, cell death, lipid metabolism and cell differentiation, directly affecting tissues, organs and even our entire system^[40]^. miRNA dysregulation has been demonstrated as a causal factor in disease progression, and miRNAs can be used as biomarkers and therapeutic targets (Paul P et al., 2018). In clinical work, we can use miRNA to monitor organ status, diagnose diseases, treat diseases, etc. Numerous studies have shown that miRNA expression is altered in IRI in organs such as the heart, brain, kidney and intestine and that differential miRNA expression occurs in HIRI. miRNA mediates HIRI, while HIRI can be alleviated by regulating miRNA expression (Cao M et al., 2021; Ingram H et al., 2022; Ye L et al., 2022). We performed miRNA expression prediction for five Hub genes (Figure 8C), further revealing the relationship between ferroptosis and HIRI, which can be used to diagnose the occurrence of ferroptosis in HIRI and may provide a new approach to mitigate HIRI in clinical work. The miRNAs we predicted have the same results as those predicted in the raw letter analysis of IRI mechanisms in coronary artery bypass grafting patients (Shen Z et al., 2019) and different ones. Among them, we predicted has-miR-128-3p, and it has been shown that inhibition of miR-128-3p by Tongxinluo protects human cardiomyocytes from ischemia/reperfusion injury *via* upregulation of p70s6k1/p-p70s6k1 (Chen G et al., 2017). We predicted hsa-miR-24-3p, and it has been shown that rosuvastatin alleviates ischemia/reperfusion injury in cardiomyocytes by downregulating hsa-miR-24-3p to target up-regulated uncoupling protein 2 (Wei W et al., 2019). We, therefore, hypothesise that there is also some benefit in alleviating ferroptosis in HIRI by regulating miRNA levels.

Although we have done a great deal of work on the relationship between HIRI and ferroptosis, we acknowledge that our study has some limitations. We have only speculated on the link between HIRI and ferroptosis in theory and have used another HIRI-related dataset for preliminary validation of this. We have not further validated our conclusions in animal experiments or clinical trials. Also, our data selection is somewhat limited in that the datasets we selected were all about HIRI in liver transplantation and not about HIRI in liver resection. Therefore, these limitations will also be the focus of our future research efforts to investigate the link between HIRI and ferroptosis in depth and to design and implement rigorous animal or clinical trials to test the theoretical speculations we have obtained. We will apply the results of our research to clinical work to reduce the risks of HIRI, improve the prognosis of patients and enhance their quality of life.

## Conclusion

Ferroptosis, a newly discovered mode of cell death, plays an essential role in HIRI. The 11 DEGs and 5 Hub genes of HIRI that we screened by the bioinformatic analysis were closely associated with ferroptosis. Further bioinformatic analysis methods of these genes are beneficial in revealing the potential biological pathways and mechanisms of ferroptosis in HIRI, providing a reference for further in-depth studies. The targeted drugs and miRNA predicted for the five Hubs provide some reference value for clinical work in assessing the diagnosis of ferroptosis in HIRI, the extent of its occurrence and the mitigation of HIRI by means of ferroptosis inhibition. The five Hub genes can be used as potential biomarkers and therapeutic targets for HIRI and also provide some theoretical basis and research directions to explore the relationship between HIRI and ferroptosis further.Oliveros, 2007, R Core Team. R, 2022.

## Data Availability

The original contributions presented in the study are included in the article/Supplementary Material, further inquiries can be directed to the corresponding author.
